# Insulin resistance and coronary inflammation in patients with coronary artery disease: a cross-sectional study

**DOI:** 10.1186/s12933-024-02159-5

**Published:** 2024-02-24

**Authors:** Tingjie Yang, Guoyong Li, Che Wang, Guian Xu, Qingman Li, Yapan Yang, Lijie Zhu, Lulin Chen, Xueqing Li, Honghui Yang

**Affiliations:** 1 Department of Cardiology, Central China Fuwai Hospital, Central China Fuwai Hospital of Zhengzhou University, Henan Provincial People’s Hospital Heart Center, Zhengzhou, 451464 Henan China; 2 Department of Cardiology, Henan Provincial People’s Hospital, People’s Hospital of Zhengzhou University, Zhengzhou, 450003 Henan China; 3 Department of Cardiology, West China Hospital, Sichuan University, Chengdu, 610041 Sichuan Province P. R. China

**Keywords:** Insulin resistance, Triglyceride-glucose index, Coronary inflammation, Peri-coronary adipose tissue attenuation, Mediation analysis

## Abstract

**Background:**

Insulin resistance (IR) is associated with coronary artery disease (CAD) severity. However, its underlying mechanisms are not fully understood. Therefore, our study aimed to explore the relationship between IR and coronary inflammation and investigate the synergistic and mediating effects of coronary inflammation on the association between IR and CAD severity.

**Methods:**

Consecutive patients with CAD who underwent coronary angiography and coronary computed tomography angiography between April 2018 and March 2023 were enrolled. The triglyceride–glucose index (TyG index) and peri-coronary adipose tissue (PCAT) attenuation around the proximal right coronary artery (RCA) were used to evaluate IR and coronary inflammation, respectively. The correlation between the TyG index and PCAT attenuation was analyzed using linear regression models. Logistic regression models were further used for investigating the correlation of the TyG index and PCAT attenuation with CAD severity. A mediation analysis assessed the correlation between IR and CAD severity mediated by coronary inflammation.

**Results:**

A total of 569 participants (mean age, 62 ± 11 years; 67.8% men) were included in the study. PCAT attenuation was positively associated with the TyG index (*r* = 0.166; *P* < 0.001). After adjusting for potential confounders, the per standard deviation increment in the TyG index was associated with a 1.791 Hounsfield unit (HU) increase (95% confidence interval [CI], 0.920–2.662 HU; *P* < 0.001) in the PCAT attenuation. In total, 382 (67.1%) patients had multivessel CAD. The patients in the high-TyG index/high PCAT attenuation group had approximately 3.2 times the odds of multivessel CAD compared with those in the low-TyG index/low PCAT attenuation group (odds ratio, 3.199; 95%CI, 1.826–5.607; *P* < 0.001). Mediation analysis indicated that PCAT attenuation mediated 31.66% of the correlation between the TyG index and multivessel CAD.

**Conclusions:**

The TyG index positively correlated with PCAT attenuation in patients with CAD. The TyG index and PCAT attenuation showed a synergistic correlation with multivessel CAD. Furthermore, PCAT attenuation partially mediated the relationship between the TyG index and CAD severity. Controlling inflammation in patients with high IR and coronary inflammation may provide additional benefits.

**Supplementary Information:**

The online version contains supplementary material available at 10.1186/s12933-024-02159-5.

## Background

Coronary artery disease (CAD) is a significant global health issue that poses substantial challenges in terms of both disability and mortality [1]. Insulin resistance (IR) has emerged as a crucial factor of interest in this regard. IR is a state of diminished responsiveness to the normal actions of insulin [2]. A growing body of evidence suggests that IR plays a pivotal role in the pathogenesis of coronary atherosclerosis [3]. High IR levels have been linked to adverse cardiovascular events [2]. The triglyceride-glucose (TyG) index, calculated using triglycerides and fasting blood glucose measurements, has been suggested as a substitute for IR [4]. Several studies have shown that an elevated TyG index is independently associated with a higher risk of multivessel CAD and worse prognosis in patients with acute coronary syndrome (ACS) [5–7]. However, its underlying mechanisms are not yet fully understood. Recent studies have indicated that one of these mechanisms might be associated with inflammation [8–10].

The development of coronary atherosclerotic plaque remodeling, which can result in plaque rupture and myocardial infarction, is heavily influenced by vascular inflammation [11]. In recent years, coronary computed tomography angiography (CCTA) has emerged as a dependable tool for evaluating coronary inflammation by analyzing changes in peri-coronary adipose tissue (PCAT) attenuation [12, 13]. Inflammation inhibits local adipogenesis in perivascular fat, changing its composition around inflamed arteries and shifting attenuation on CCTA from the lipid (more negative Hounsfield unit [HU] values [closer to − 190 HU]) to the aqueous phase (less negative HU values [closer to − 30 HU]). PCAT attenuation has demonstrated great potential in distinguishing between different stages of coronary artery disease (CAD) [14] and identifying culprit lesions in patients with ACS [15]. In addition, Oikonomou et al. demonstrated that PCAT attenuation of the right coronary artery (RCA) is a representative biomarker for global coronary inflammation and identifies individuals at risk of all-cause and cardiac mortality over traditional cardiovascular risk factors [13].

Although previous studies have acknowledged the roles of IR and inflammation in atherosclerosis development, there are limited comprehensive investigations into the causal pathways that link between IR, coronary inflammation, and CAD severity. Some studies have reported that IR is positively associated with high-sensitivity C-reactive protein (hs-CRP), reflecting systemic inflammation [2, 16]. Li et al. have further demonstrated that IR and hs-CRP together increased cardiovascular risk and that hs-CRP partly mediates the association between IR and clinical outcomes in patients with diabetic chronic coronary syndrome [10]. However, highlighting that hs-CRP is not directly associated with coronary atherogenesis and has weak specificity for coronary inflammation is crucial [17].

Therefore, the present study aimed to assess the relationship between TyG index-reflected IR and PCAT attenuation-reflected coronary inflammation in patients with CAD and the impact of glucose metabolic states on this association. In addition, we evaluated the combined and mediating effects of PCAT attenuation on the correlation between the TyG index and CAD severity. Conducting this research, we opted for gaining a better understanding of the complex interplay among insulin resistance, coronary inflammation, and CAD severity.

## Methods

### Study population

**Fig. 1:**
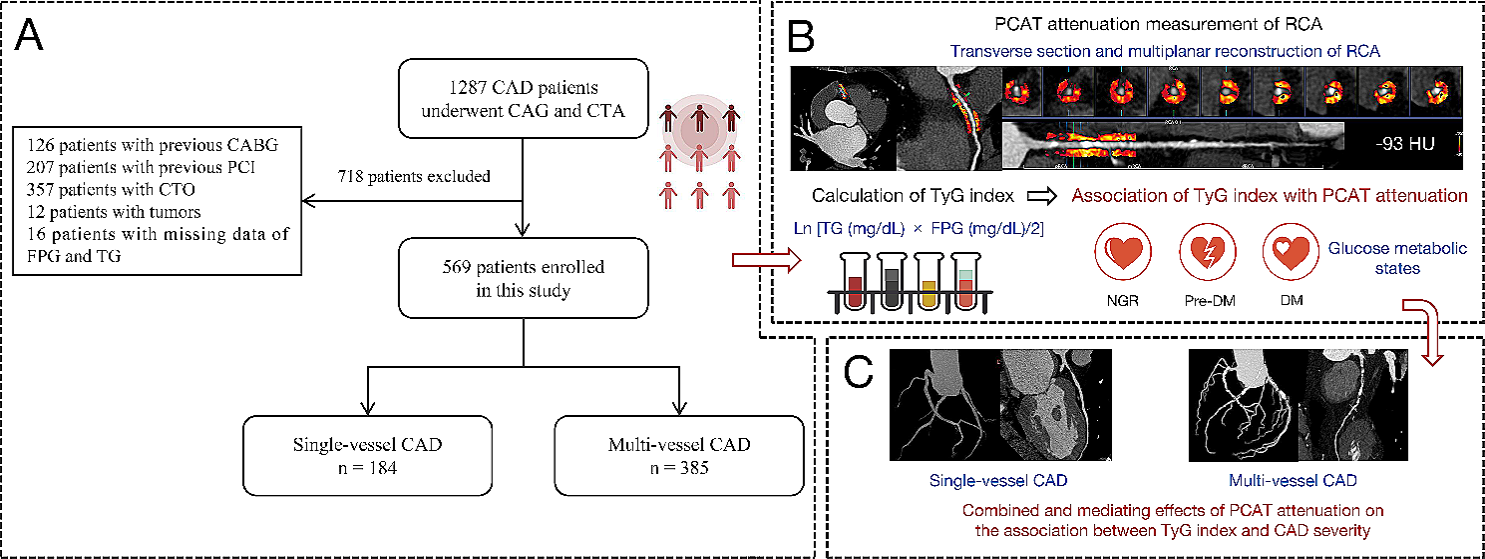
Flowchart of patient recruitment and study design. CAD, coronary artery disease; CAG, coronary angiography; CTA, computed tomography angiography; CABG, coronary artery bypass grafting; PCI, percutaneous coronary intervention; CTO, chronic total occlusion; FPG, fasting plasma glucose; TG, triglyceride; PCAT, peri-coronary adipose tissue; RCA, right coronary artery; TyG, triglyceride-glucose

This study enrolled 1287 consecutive patients with CAD who underwent CCTA and coronary angiography (CAG) between April 2018 and March 2023 at the Central China Fuwai Hospital. We excluded patients: (1) younger than 25 years or older than 95 years; (2) with tumors or severe liver or kidney disease; (3) who lacked data on triglyceride (TG) and fasting plasma glucose (FPG); (4) with a previous history of percutaneous coronary intervention (PCI) or coronary artery bypass grafting (CABG); (5) with coronary chronic total occlusion (CTO); and (6) with poor CCTA image quality due to respiratory motion artifact and irregular heart rates. Finally, 569 patients were included in this study. A flowchart of patient recruitment and research design is illustrated in Fig. 1.

The study was conducted in accordance with the principles expressed in the Declaration of Helsinki and approved by the Ethics Committee of Central China Fuwai Hospital. Written informed consent was obtained from all participants.

### Data collection and definitions

All anthropometric parameters, clinical history, and laboratory test results were acquired from electronic medical records. The anthropometric characteristics included age, sex, height, weight, blood pressure, and smoking status. The clinical history included chronic kidney disease, cancer, diabetes, and hypertension. Antiplatelet, antilipidemic, antihypertensive, and antidiabetic medications have also been recorded.

Blood samples were collected from all participants after at least 8 h of fasting. Laboratory parameters, including FPG, TG, total cholesterol (TC), low-density lipoprotein-C (LDL-C), high-density lipoprotein-C (HDL-C), glycated hemoglobin (HbA1c), and serum creatinine, were measured by standard biochemical techniques in the experimental medicine department. Body mass index (BMI) was calculated as weight (kg) divided by height squared (m^2^). The TyG index was arithmetically derived as follows: ln [TG (mg/dL) × FPG (mg/dL)/2].

The CAG was performed by professionals blinded to the study protocol. In addition, at least two experienced cardiologists reviewed the coronary angiography results. CAD was referred to as at least one major coronary artery with ≥ 50% stenosis. The number of coronary arteries with ≥ 50% stenosis indicated the CAD severity. The multi-vessel CAD was defined as two or more main epicardial coronary artery stenoses ≥ 50%.

According to the American Diabetes Association criteria [18], diabetes mellitus (DM) is characterized as FPG ≥ 7.0 mmol/L, 2-h plasma glucose level ≥ 11.1 mmol/L according to the oral glucose tolerance test, or HbA1c ≥ 6.5%. Pre-DM was diagnosed when individuals had an FPG ranging between 5.6 and 6.9 mmol/L, 2-h plasma glucose level ranging between 7.8 and 11.0 mmol/L, or HbA1c level ranging between 5.7% and 6.4%. Normal glucose regulation (NGR) was defined as FPG < 5.6 mmol/L or HbA1c < 5.7%.

### CCTA imaging protocol

CCTA was performed using a third-generation dual-source CT scanner (Somatom Force; Siemens Healthineers, Forchheim, Germany). All images were acquired using prospective ECG gating. A beta-blocker was administered orally to achieve a heart rate ≤ 65 beats/min. The iodinated contrast (iopromidum; Bayer Pharmaceuticals, Berlin, Germany; 50–60 mL, 370 mg iodine/mL) was injected at a flow rate of 4.5 mL/s. Scanning was performed with an automatic tube potential selection (CARE kV, Siemens Healthineers; reference, 100 kV) and tube current modulation (CARE Dose 4D, Siemens Healthineers; reference, 320 mAs) technique adjusted to patient’s body habitus and a 0.25-s gantry rotation time. Images were reconstructed with a 512 × 512 matrix, 0.75 mm slice thickness, and 0.5 mm increments.

### PCAT attenuation measurement

Previous research described PCAT attenuation around the proximal RCA as a representative indicator of global coronary inflammation [13]. To eliminate the impact of the aortic wall, we focused on the proximal 10–50 mm of the RCA for our per-patient PCAT attenuation analysis, which was automatically performed using a Perivascular Fat Analysis Tool (Shukun Technology). In brief, adipose tissue was defined as all voxels in the HU range between − 190 and − 30 HU located within a radial distance from the outer vessel border equal to the diameter of the surrounding vessel. PCAT attenuation was then calculated as the average attenuation of the adipose tissue surrounding the vessel.

### Statistical analysis

Normally and non-normally distributed continuous data are expressed as median (interquartile range) and mean ± standard deviation (SD), respectively, and categorical variables are expressed as absolute values (percentages). Continuous variables were compared using a one-way analysis of variance or the Kruskal-Wallis test. Categorical variables were compared using the Pearson chi-square or the Fisher exact test.

The correlation between the TyG index and PCAT attenuation was evaluated using linear regression models. Model 1 was unadjusted. Model 2 was adjusted for age and sex. Model 3 was adjusted for the variables in model 2 and further adjusted for BMI; smoking; hypertension; estimated glomerular filtration rate (eGFR); and antiplatelets, statins, ACEI/ARB, beta-blockers, and antidiabetic drug use. Restricted cubic spline regression was used to visually model the connection between the TyG index as a continuous variable and PCAT attenuation. To analyze the association of the TyG index and PCAT attenuation with CAD severity (single-vessel CAD versus multi-vessel CAD), odds ratios (ORs) and 95% confidence intervals (CIs) were calculated using logistic regression models.

The mediation package in the R software was used for mediation analysis to assess whether PCAT attenuation mediated the association between IR and CAD severity [19]. This study used a directed acyclic graph to visualize the assumed causal model with the TyG index (continuous) as the exposure, PCAT attenuation (continuous) as the mediator, and multivessel CAD as the outcome variable. Confounders identified using directed acyclic graphs were adjusted for. The significance of the mediating effect was examined using 1000 bootstrap samples. A *P* value < 0.05 was considered statistically significant, and statistical analysis was performed using R software (version 4.3.1).

## Results

### Demographic and clinical characteristics

The average age of the 569 participants was 62 ± 11 years; 67.8% were men. The mean TyG index was 8.79 ± 0.69. The mean PCAT attenuation was − 83.5 ± 10.4 HU. Table 1 presents the characteristics based on the tertiles of the TyG index.

**Table 1:** Demographic and clinical characteristics

Characteristics	Total patients (*n* = 569)	TyG index (in tertiles)			*P* value
		T1 group (TyG index < 8.50)	T2 group (8.50 ≤ TyG index ≤ 8.98)	T3 group (TyG index > 8.98)	
Age, years	62 ± 11	63 ± 11	63 ± 10	59 ± 11^**‡‡^	< 0.001
Male, n (%)	386 (67.8)	129 (66.8)	130 (68.4)	127 (68.3)	0.935
BMI, kg/m^2^	25.70 ± 3.29	24.71 ± 3.04	25.91 ± 3.04^**^	26.52 ± 3.54^**^	< 0.001
SBP, mmHg	138 ± 19	137 ± 18	139 ± 20	139 ± 21	0.469
DBP, mmHg	85 ± 13	84 ± 13	85 ± 13	88 ± 14^*^	0.017
HR, bpm	76 ± 12	75 ± 13	75 ± 11	78 ± 12	0.027
Smoking, n(%)	215 (37.8)	66 (34.2)	73 (38.4)	76 (40.9)	0.399
Hypertension, n(%)	353 (62.0)	110 (57.0)	124 (65.3)	119 (64.0)	0.200
Glucose metabolism state, n(%)					< 0.001
Normoglycemia	241 (42.4)	120 (62.2)	74 (38.9)^*^	47 (25.3)^*‡^	
Pre-diabetes mellitus	88 (15.5)	32 (16.6)	33 (17.4)^*^	23 (12.4)^*‡^	
Diabetes mellitus	240 (42.2)	41 (21.2)	83 (43.7)^*^	116 (62.4)^*‡^	
**Serum biomarkers**					
eGFR, mL/(min×1.73m^2^)	84.84 ± 16.96	84.12 ± 15.92	83.51 ± 17.50	86.94 ± 17.35	0.113
FPG, mmol/L	6.20 ± 2.54	5.05 ± 1.36	5.77 ± 1.70^**^	7.83 ± 3.27^**‡‡^	< 0.001
Total cholesterol, mg/dL	158.82 ± 40.83	146.07 ± 33.87	159.93 ± 41.55^**^	170.93 ± 42.97^**‡^	< 0.001
HDL-C, mg/dL	41.79 ± 10.67	45.07 ± 10.49	42.04 ± 11.01^*^	38.13 ± 9.32^**‡‡^	< 0.001
LDL-C, mg/dL	95.94 ± 34.49	87.59 ± 29.70	100.16 ± 37.34^**^	100.29 ± 34.66^**^	< 0.001
Triglyceride, mg/dL	141.26 ± 75.72	89.94 ± 33.34	131.02 ± 30.35^**^	206.74 ± 92.38^**‡‡^	< 0.001
**Medications**, n(%)					
Antiplatelets	145 (25.5)	51 (26.4)	48 (25.3)	46 (24.7)	0.928
Statins	149 (26.2)	56 (29.0)	48 (25.3)	45 (24.2)	0.531
ACEIs/ARBs	114 (20.0)	37 (19.2)	37 (19.5)	40 (21.5)	0.828
Beta-blockers	91 (16.0)	27 (14.0)	35 (18.4)	29 (15.6)	0.489
Antidiabetic drugs	95 (16.7)	18 (9.3)	35 (18.4)^*^	42 (22.6)^*^	0.002

TyG, triglyceride-glucose; BMI, body mass index; SBP, systolic blood pressure; DBP, diastolic blood pressure; HR, heart rate; eGFR, estimated glomerular filtration rate; FPG, fasting plasma glucose; HDL-C, high-density lipoprotein cholesterol; LDL-C, low-density lipoprotein cholesterol; ACEI, angiotensin-converting enzyme inhibitor; ARB, angiotensin receptor blocker

^*^ adjusted *P* < 0.05 versus low TyG index; ^**^ adjusted *P* < 0.01 versus low TyG index; ^‡^ adjusted *P* < 0.05 versus moderate TyG index; ^‡‡^ adjusted *P* < 0.01 versus moderate TyG index

Compared with the lowest TyG index tertile (T1) group, the participants in the highest TyG index tertile (T3) tended to be younger and had higher BMI and FPG, LDL-C, TC, and TG levels (all, *P* < 0.05). In addition, the proportion of patients with DM and antidiabetic drugs use were higher in the T3 group than in the T1 group (both, *P* < 0.05).

### Association of TyG index with PCAT attenuation

**Fig. 2:**
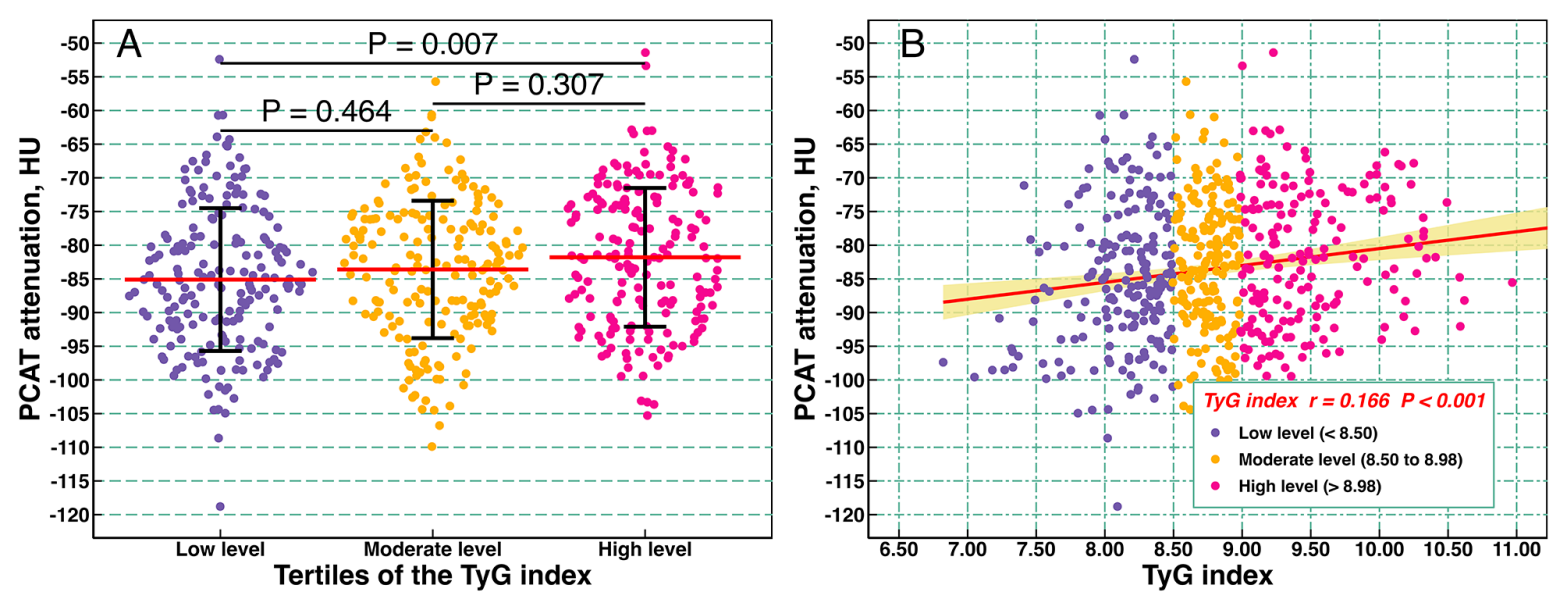
Association between TyG index and PCAT attenuation. (**A**) Violin plot showing the distribution of PCAT attenuation among groups categorized by TyG index tertiles; (**B**) scatter plot. TyG, triglyceride-glucose; PCAT, peri-coronary adipose tissue

The PCAT attenuation increased with increasing tertiles of TyG index, and the PCAT attenuation for each TyG index tertile groups were − 85.1 ± 10.6, -83.6 ± 10.2, and − 81.8 ± 10.3, respectively (Fig. 2A). Overall, PCAT attenuation was positively associated with the TyG index (*r* = 0.166; *P* < 0.001) (Fig. 2B). Regarding linear regression models measuring the TyG index as a continuous variable, each SD increment in the TyG index was associated with a 1.791 HU increase (95% CI, 0.920–2.662 HU; *P* < 0.001) in PCAT attenuation after adjusting for age; sex; BMI; smoking; hypertension; eGFR; and antiplatelets, statins, ACEI/ARB, beta-blockers, and antidiabetic drug use (Tables 2and Additional File 2: Table S1). Likewise, the categorical analysis revealed that, compared with the T1 group, the T3 group was significantly associated with a 3.144 HU (95% CI, 1.016–5.271 HU; *P* = 0.004) increase in PCAT attenuation after adjusting for all covariates.

**Fig. 3:**
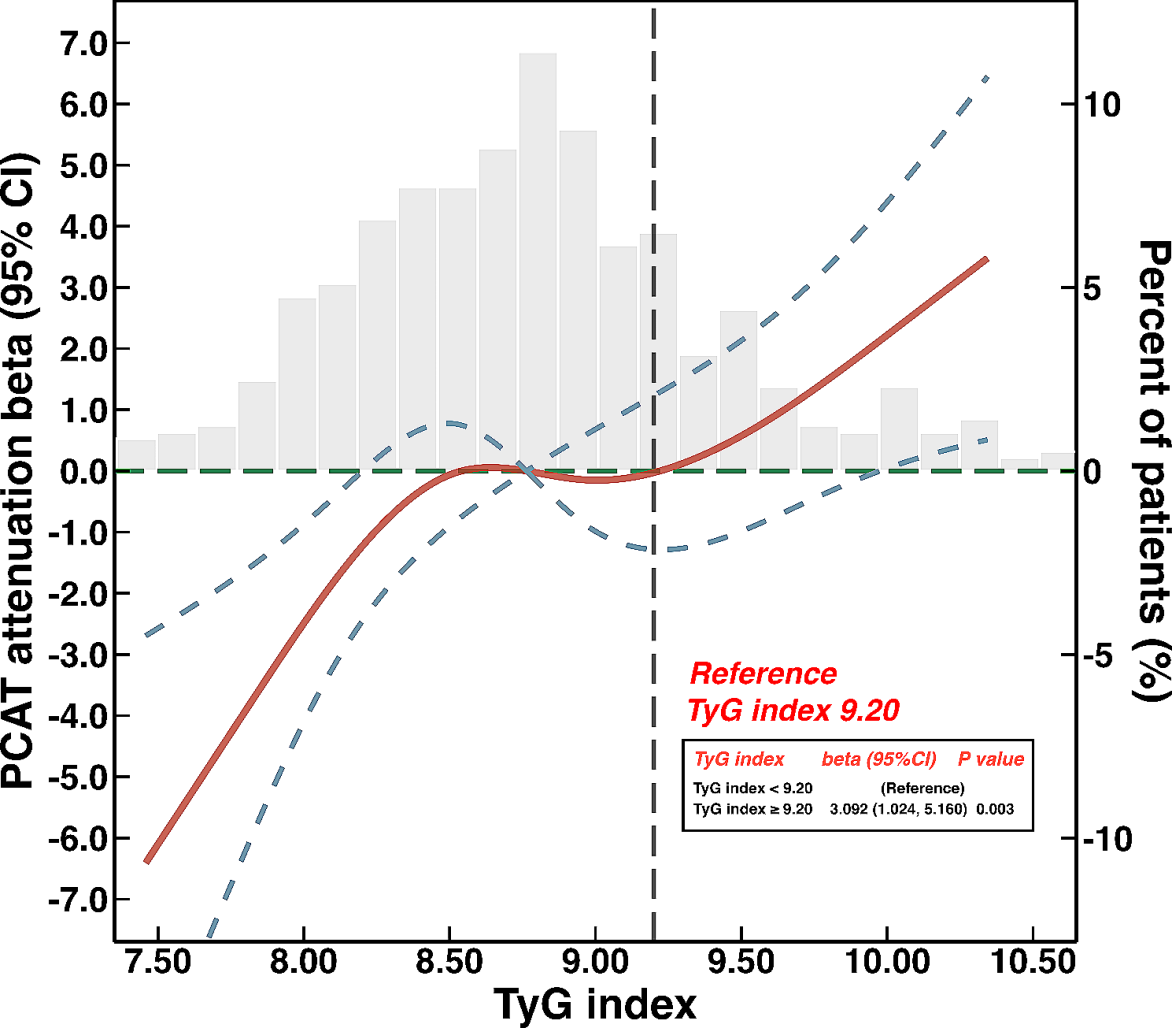
Restricted cubic splines to flexibly model and visualize the relation of TyG index with PCAT attenuation. Red lines represent the estimated β, with light blue dashed lines denoting 95% confidence intervals (CIs). The background histograms (light gray color) represent the percent of the density distribution of the TyG index in the study population (right y-axis). The vertical gray dotted lines indicate the TyG index’s threshold value at 9.20. The horizontal green dotted lines represent the β of 0. TyG, triglyceride-glucose; PCAT, peri-coronary adipose tissue; CI, confidence interval

We used restricted cubic splines to flexibly model and visualize the relationship between the TyG index and PCAT attenuation, as shown in Fig. 3. After adjusting for potential confounders, we identified a dose-response relationship between the TyG index and PCAT attenuation (nonlinear; *P* = 0.109). A cut-off value of 9.20 was chosen to split the entire patients, and patients with TyG index ≥ 9.20 exhibited significantly higher PCAT attenuation compared with those with TyG index < 9.20 (β, 3.092; 95% CI, 1.024–5.160; *P* = 0.003).

**Table 2:** Association between TyG index and PCAT attenuation

Characteristics	Model 1		Model 2		Model 3	
	β (95% CI)	*P* value	β (95% CI)	*P* value	β (95% CI)	*P* value
TyG index (per SD)	1.730 (0.884, 2.576)	< 0.001	1.916 (1.066, 2.765)	< 0.001	1.791 (0.920, 2.662)	< 0.001
TyG index (tertiles)						
T1 group (< 8.50)	1 (Reference)		1 (Reference)		1 (Reference)	
T2 group (8.50 to 8.98)	1.511 (-0.562, 3.584)	0.153	1.516 (-0.530, 3.563)	0.146	1.063 (-0.992, 3.118)	0.311
T3 group (> 8.98)	3.262 (1.178, 5.346)	0.002	3.572 (1.487, 5.657)	0.001	3.144 (1.016, 5.271)	0.004
*P* value for trend	0.002		0.001		0.004	

Model 1: unadjusted;

Model 2: adjusted for age and sex;

Model 3: adjusted for age, sex, BMI, smoking, hypertension, eGFR, antiplatelet drug use, statin drug use, ACEI/ARB drug use, beta-blocker drug use, and antidiabetic drug use

TyG, triglyceride-glucose; PCAT, pericoronary adipose tissue; CI, confidence interval; SD, standard deviation; BMI, body mass index; eGFR, estimated glomerular filtration rate; ACEI, angiotensin-converting enzyme inhibitor; ARB, angiotensin receptor blocker

Tables 3 and Additional File 2: Table S2 present the relationship between the TyG index and PCAT attenuation according to different diabetes states, including NGR, pre-DM, and DM. Adjusting for age, sex, BMI, smoking, hypertension, eGFR, and medication usage in model 3, the TyG index as a continuous variable was independently correlated with PCAT attenuation in the subgroups of pre-DM (β, 2.652; 95% CI, 0.060–5.245; *P* = 0.045) and DM (β, 1.825; 95% CI, 0.643–3.007; *P* = 0.002). For the categorical analysis, in the pre-DM (β, 5.718; 95% CI, 1.066–10.370; *P* = 0.016) and DM (β, 4.112; 95% CI, 1.041–7.184; *P* = 0.009) subgroups, the highest tertile of TyG index was associated with a significantly higher PCAT attenuation when the lowest tertile of TyG index was used as the reference after adjusting for confounding factors.

**Table 3:** Associations between TyG index and PCAT attenuation according to different diabetes statuses

Characteristics	Model 1		Model 2		Model 3	
	β (95% CI)	*P* value	β (95% CI)	*P* value	β (95% CI)	*P* value
Normoglycemia (*n* = 241)						
TyG index (per SD)	1.679 (-0.114, 3.473)	0.067	2.038 (0.279, 3.798)	0.023	1.405 (-0.401, 3.211)	0.127
TyG index (tertiles)						
Low level	1 (Reference)		1 (Reference)		1 (Reference)	
Moderate level	3.204 (-0.280, 6.688)	0.072	3.386 (0.010, 6.762)	0.049	2.437 (-0.914, 5.789)	0.154
High level	0.622 (-2.862, 4.106)	0.726	1.026 (-2.413, 4.465)	0.559	0.087 (-3.367, 3.541)	0.961
*P* value for trend	0.797		0.598		0.932	
Pre-diabetes mellitus (*n* = 88)						
TyG index (per SD)	3.157 (0.795, 5.519)	0.009	3.089 (0.531, 5.646)	0.018	2.652 (0.060, 5.245)	0.045
TyG index (tertiles)						
Low level	1 (Reference)		1 (Reference)		1 (Reference)	
Moderate level	5.308 (0.871, 9.745)	0.019	5.439 (1.038, 9.840)	0.015	4.385 (-0.059, 8.830)	0.053
High level	6.339 (1.940, 10.739)	0.005	6.691 (2.082, 11.300)	0.004	5.718 (1.066, 10.370)	0.016
*P* value for trend	0.005		0.005		0.015	
Diabetes mellitus (*n* = 240)						
TyG index (per SD)	1.743 (0.613, 2.874)	0.002	1.930 (0.750, 3.109)	0.001	1.825 (0.643, 3.007)	0.002
TyG index (tertiles)						
Low level	1 (Reference)		1 (Reference)		1 (Reference)	
Moderate level	2.332 (-0.719, 5.384)	0.134	2.407 (-0.641, 5.455)	0.122	2.155 (-0.899, 5.208)	0.167
High level	3.992 (0.950, 7.034)	0.010	4.265 (1,161, 7.369)	0.007	4.112 (1.041, 7.184)	0.009
*P* value for trend	0.011		0.008		0.009	

Model 1: unadjusted;

Model 2: adjusted for age and sex;

Model 3: adjusted for age, sex, BMI, smoking, hypertension, eGFR, antiplatelet drug use, statin drug use, ACEI/ARB drug use, beta-blocker drug use, and antidiabetic drug use

TyG, triglyceride-glucose; PCAT, pericoronary adipose tissue; CI, confidence interval; SD, standard deviation; BMI, body mass index; eGFR, estimated glomerular filtration rate; ACEI, angiotensin-converting enzyme inhibitor; ARB, angiotensin receptor blocker

### Combined and mediating effects of PCAT attenuation on the relation of TyG index with CAD severity

Among the 569 enrolled participants, 382 (67.1%) had multi-vessel CAD. The relationship between multivessel CAD and the TyG index or PCAT attenuation is depicted in Additional File 1: Fig. S1. The logistic regression analysis of multivessel CAD is shown in Additional File 2: Table S3. After adjusting for all confounders, the odds of multi-vessel CAD increased rapidly and remained relatively flat until a TyG index of 8.59. A cut-off value of 8.59 was chosen to divide the entire patients, and patients with TyG index ≥ 8.59 were significantly correlated with increased odds of multi-vessel CAD compared with those with TyG index < 8.59 (OR, 1.692; 95% CI, 1.158–2.474; *P* = 0.007) based on adjusted multivariable logistic regression analysis. Likewise, the total patients were divided into two categories according to the cut-off value of PCAT attenuation (-80.9 HU). Regarding the fully adjusted regression model, patients with PCAT attenuation ≥ -80.9 HU were significantly correlated with CAD severity compared with those with PCAT attenuation < -80.9 HU (OR, 1.876; 95% CI, 1.271–2.769; *P* = 0.002).

When patients were categorized by the combination of TyG index and PCAT attenuation and adopting the L-TyG index/L-PCAT attenuation group as a reference, we found that the H-TyG index/H-PCAT attenuation group had approximately 3.2 times the odds of multivessel CAD (OR, 3.199; 95% CI, 1.826–5.607; *P* < 0.001) after adjusting for all covariates (Table 4). However, CAD severity did not significantly increase in the L-TyG index/H-PCAT attenuation or the H-TyG index/L-PCAT attenuation groups.

**Fig. 4:**
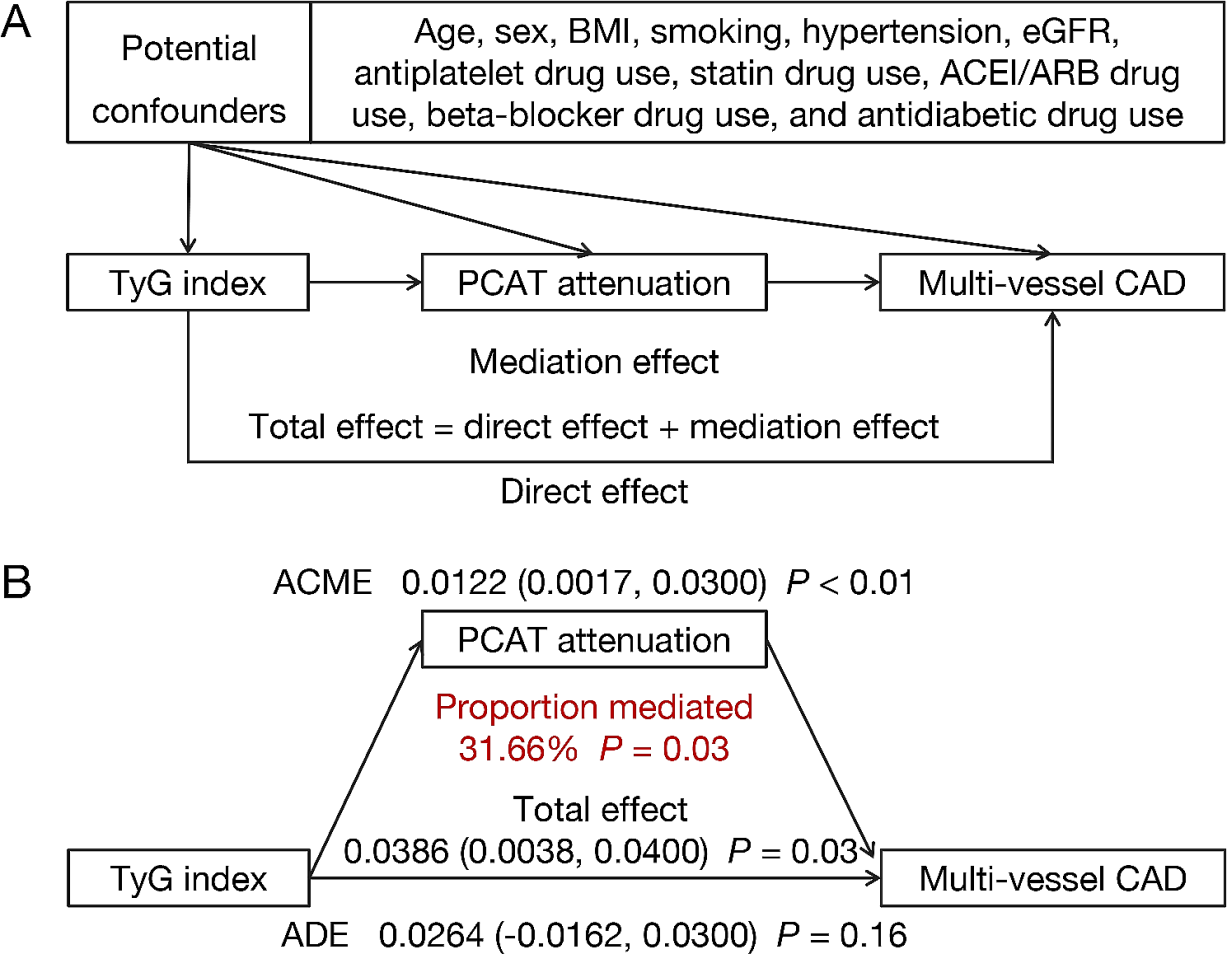
Mediation effect of PCAT attenuation on the association between TyG index and multi-vessel CAD. (**A**) Directed acyclic graph; (**B**) mediation analysis of multi-vessel CAD. CAD, coronary artery disease; ACME, average causal mediation effect; ADE, average direct effect; PCAT, peri-coronary adipose tissue; TyG, triglyceride-glucose; BMI, body mass index; eGFR, estimated glomerular filtration rate; ACEI, angiotensin converting enzyme inhibitor; ARB, aldosterone receptor blocker

A mediation analysis was performed to investigate the mediating effects of PCAT attenuation. As illustrated in Fig. 4, mediation analysis indicated a significant partial mediating effect of coronary inflammation on the relationship between IR and CAD severity. In particular, PCAT attenuation mediated 31.66% of the correlation between the TyG index and multivessel CAD.

**Table 4:** Association of TyG index and PCAT attenuation with severity of CAD

Characteristics	Model 1		Model 2		Model 3	
	OR (95% CI)	*P* value	OR (95% CI)	*P* value	OR (95% CI)	*P* value
L-TyG index/L-PCAT attenuation	1 (Reference)		1 (Reference)		1 (Reference)	
L-TyG index/H-PCAT attenuation	1.394 (0.791, 2.459)	0.251	1.252 (0.703, 2.230)	0.445	1.281 (0.711, 2.307)	0.410
H-TyG index/L-PCAT attenuation	1.369 (0.882, 2.126)	0.162	1.423 (0.910, 2.226)	0.122	1.329 (0.835, 2.116)	0.231
H-TyG index/H-PCAT attenuation	3.621 (2.117, 6.195)	< 0.001	3.579 (2.079, 6.161)	< 0.001	3.199 (1.826, 5.607)	< 0.001
*P* value for trend	< 0.001		< 0.001		< 0.001	
*P* value for interaction	0.101		0.077		0.116	

Model 1: unadjusted;

Model 2: adjusted for age and sex;

Model 3: adjusted for age, sex, BMI, smoking, hypertension, eGFR, antiplatelet drug use, statin drug use, ACEI/ARB drug use, beta-blocker drug use, and antidiabetic drug use

PCAT, pericoronary adipose tissue; CAD, coronary artery disease; L, low; H, high; TyG, triglyceride-glucose; OR, odds ratio; CI, confidence interval; BMI, body mass index; eGFR, estimated glomerular filtration rate; ACEI, angiotensin-converting enzyme inhibitor; ARB, angiotensin receptor blocker

## Discussion

IR is one of the most significant risk factors for development and progression of CAD [20, 21]. Our findings confirmed that the TyG index was related to disease severity in individuals with CAD, consistent with prior research [22]. Furthermore, the present study showed that the TyG index positively correlated with PCAT attenuation around the proximal RCA, which remained in the pre-DM and DM subgroups. The TyG index and PCAT attenuation showed a synergistic correlation with CAD severity. In addition, the mediation analysis indicated that PCAT attenuation partially mediated the relationship between the TyG index and multivessel CAD.

IR can decrease lipoprotein lipase activity in adipocytes, resulting in an increased production of free fatty acids (FFA) and inflammatory cytokines, such as interleukin (IL)-6, tumor necrosis factor alpha (TNF-α), and leptin [2]. Additionally, FFA imposes a direct influence on transcription factors and upregulates the IKK/NF-κB inflammatory signaling pathway, which would lead to the further activation of TNF-α, IL-1β, and IL-6, and elevated plasma levels of the monocyte chemotactic protein-1 (MCP-1) [23]. IR can also activate the NLRP3 inflammasome, which triggers the cleavage of pro-IL-1β and pro-IL-18 and exacerbates subsequent inflammatory cascades [24].

Several epidemiological studies have also indicated the pro-inflammatory characteristics of IR. Srilatha et al. discovered a positive association between IR and hs-CRP in a cohort of first-degree relatives of patients with ischemic stroke [25]. Similarly, Jin et al. observed that IR was associated with hs-CRP in nondiabetic individuals with ischemic stroke [16]. In contrast to these studies, we assessed CT attenuation of PCAT as a specific biomarker for coronary inflammation. Based on the available evidence, the present study is the first to observe a dose-response relationship between the TyG index and coronary inflammation. However, this association was not observed in patients with normal glucose levels. It is speculated that the probable mechanism of coronary inflammation may be more related to other risk factors, such as dyslipidemia and oxidative stress, than to IR, in these patients. However, the accuracy of this hypothesis should be confirmed in future studies. The residual burden of atherosclerotic cardiovascular disease remains large in patients with abnormal glucose metabolism, despite using guideline-based preventive medications. Our study found that the TyG index correlated with coronary inflammation in patients with pre-DM or DM. This suggests that coronary inflammation might be a residual risk in these patients and anti-inflammatory therapies may offer an opportunity to further reduce the burden of atherosclerotic cardiovascular disease risk. In terms of disease prevention, a patient screening taking PCAT attenuation into account might help with an earlier identification of severe CAD.

IR initiates the inflammatory response and progression of atherosclerosis, which supports the hypothesis that inflammation may mediate the association between IR and CAD severity. The present study found that PCAT attenuation partially mediated the TyG index and multivessel CAD relationship. Several signaling pathways may be involved in this process, including the proprotein convertase subtilisin/kexin type 9, Notch and Wnt signaling pathways, NLRP3 inflammasome, and toll-like receptors [26]. However, our study indicated that PCAT attenuation only mediated 31.66% of the relationship between the TyG index and CAD severity, implying that other mediators, such as oxidative stress, might be involved in the link. Further experimental studies are required to elucidate the mechanisms underlying the association between IR and CAD severity.

The present study showed that the combination of the TyG index and PCAT attenuation assisted in identifying individuals with greater odds of multivessel CAD, implying the combined impact of IR and coronary inflammation in promoting atherosclerosis. Endothelial integrity is essential for maintaining vascular homeostasis and inhibiting atherosclerosis development. In an IR milieu, hyperglycemia enhances leucocyte adhesion to endothelial cells, impairs endothelial function [27, 28], and aggravates inflammatory response beneath the endothelium [29]. Thus, in the presence of IR, endothelial cells may become more responsive to pro-inflammatory stimuli. Moreover, IR and inflammation can initiate and intensify each other in a vicious cycle [30], further strengthening the concept that IR and inflammation may synergistically contribute to the pathogenesis of coronary atherogenesis.

Despite current preventive efforts and effective revascularization interventions, the residual risk in patients with evident atherosclerosis remains unacceptably high [31]. The use of anti-inflammatory medications may further reduce the risk of residual inflammation. In the CANTOS trial, canakinumab, a monoclonal antibody targeting the IL-1β innate immunity pathway, significantly reduced the incidence of recurrent cardiovascular events in patients with myocardial infarction [32]. However, canakinumab is associated with a high incidence of fatal infections. Colchicine, extracted initially from autumn croci, is a potent oral anti-inflammatory agent. In the COLCOT trial, in patients with recent myocardial infarction, anti-inflammatory therapy with low-dose colchicine reduced the risk of ischemic cardiovascular events compared with placebo [33]. However, Tong et al. reported that adding colchicine to standard medical therapy did not significantly affect the cardiovascular outcomes at 12 months in patients with ACS and was associated with a higher mortality rate [34]. Therefore, the primary challenge is to identify the patients who would benefit the most from anti-inflammatory therapy. This study revealed that combining the TyG index and PCAT attenuation could assist in identifying patients with multivessel CAD. Anti-inflammatory therapies may provide additional benefits to these individuals.

### Study limitations

This study has several limitations. First, this was a cross-sectional, single-center study. Hence, we could not establish a causal relationship between IR, inflammation, and CAD severity. Our study sample size was relatively small. Moreover, the correlation coefficient between PCAT attenuation and TyG index may have been influenced by the sample size. We identified a nearly curvilinear relationship between TyG index and PCAT attenuation with a non-linear *P* value approaching a level of significance. As a statistical measure of the strength of a linear relationship between two variables, the small correlation coefficient may have not reflected the actual relationship between PCAT attenuation and TyG index. Second, patients without data on the TyG index and PCAT attenuation were excluded, possibly leading to a selection bias. Third, the metabolic control and patient treatment adherence play key roles in the management of CAD or type 2 DM, which might cause confounding effects. In the future, we will perform further longitudinal studies to adjust for the effect of patient treatment adherence and metabolic control. Fourth, our research did not calculate the IR index using the homeostasis model assessment of IR (HOMA-IR). Therefore, we could not compare the role of TyG index with the HOMA-IR in this study. We will compare the TyG index with the HOMA-IR in future studies. Finally, the acquisition tube voltages in the current study were heterogeneous, which may have affected our results [35]. However, further experimental studies are required to confirm these findings.

## Conclusions

In patients with CAD, the TyG index was associated with PCAT attenuation around the proximal RCA, which persisted in the pre-DM and DM subgroups. The TyG index and PCAT attenuation showed a synergistic correlation with multivessel CAD. Additionally, PCAT attenuation partially mediated the association between the TyG index and CAD severity. Combining the TyG index and PCAT attenuation could assist in identifying patients with severe CAD, and controlling inflammation in those with higher IR and coronary inflammation might reduce cardiovascular events and improve quality of life.

### Electronic supplementary material

Below is the link to the electronic supplementary material.

**Additional file 1**: Fig. S1. Restricted cubic splines to flexibly model and visualize the relation of TyG index (**A**) and PCAT attenuation (**B**) with multi-vessel CAD. CAD, coronary artery disease; OR, odds ratio; CI, confidence interval; TyG, triglyceride-glucose; PCAT, peri-coronary adipose tissue.

**Additional file 2**: Table S1. Association between TyG index and PCAT attenuation. Table S2. Associations between TyG index and PCAT attenuation according to different diabetes statuses. Table S3. Association of TyG index or PCAT attenuation with severity of CAD

## Data Availability

The datasets generated and analysed during the current study are not publicly available due privacy and ethical restrictions but are available from the corresponding author on reasonable request.

## Abbreviations

HOMA-IR: Homeostasis model assessment of IR
IR: Insulin resistance
IL: Interleukin
CAD: Coronary artery disease
DM: Diabetes mellitus
TyG: Triglyceride–glucose
PCAT: Peri-coronary adipose tissue
RCA: Right coronary artery
ACS: Acute coronary syndrome
CCTA: Coronary computed tomography angiography
hs-CRP: High-sensitivity C-reactive protein
PCI: Percutaneous coronary intervention
CABG: Coronary artery bypass grafting
CTO: Chronic total occlusion
TC: Total cholesterol
LDL-C: Low-density lipoprotein-C
HDL-C: High-density lipoprotein-C
HbA1c: Glycated hemoglobin
HU: Hounsfield unit
BMI: Body mass index
MCP-1: Monocyte chemotactic protein-1
TNF-α: Tumor necrosis factor alpha
CI: Confidence interval
