# Supplementary material for: Insulin resistance and coronary inflammation in patients with coronary artery disease: a cross-sectional study
Source: Cardiovasc Diabetol. 2024 Feb 24;23:79. doi: 10.1186/s12933-024-02159-5 (PMC10893710; doi:10.1186/s12933-024-02159-5)
Supplement: Supplementary file 2 — Additional file 2: Table S1. Association between TyG index and PCAT attenuation. Table S2. Associations between TyG index and PCAT attenuation according to different diabetes statuses. Table S3. Association of TyG index or PCAT attenuation with severity of CAD [file 12933_2024_2159_MOESM2_ESM.docx]

| **Additional File 2: Table S1 Association between TyG index and PCAT attenuation** | | | | | | |
| --- | --- | --- | --- | --- | --- | --- |
| **Characteristics** | Model 1 | | Model 2 | | Model 3 | |
|  | β (95% CI) | *P* value | β (95% CI) | *P* value | β (95% CI) | *P* value |
| TyG index (per SD) | 1.730 (0.884, 2.576) | < 0.001 | 1.916 (1.066, 2.765) | < 0.001 | 1.791 (0.920, 2.662) | < 0.001 |
| TyG index (median)* |  | |  | |  |  |
| Low level (< 8.77) | 1 (Reference) | | 1 (Reference) | | 1 (Reference) | |
| High level (≥ 8.77) | 1.856 (0.148, 3.564) | 0.033 | 2.042 (0.342, 3.742) | 0.019 | 1.943 (0.220, 3.667) | 0.027 |
| * TyG index was converted into a binary variable according to the median value.  Model 1: unadjusted;  Model 2: adjusted for age and sex;  Model 3: adjusted for age, sex, BMI, smoking, hypertension, eGFR, antiplatelet drug use, statin drug use, ACEI/ARB drug use, beta-blocker drug use, and antidiabetic drug use.  TyG, triglyceride-glucose; PCAT, pericoronary adipose tissue; CI, confidence interval; SD, standard deviation; BMI, body mass index; eGFR, estimated glomerular filtration rate; ACEI, angiotensin-converting enzyme inhibitor; ARB, angiotensin receptor blocker. | | | | | | |

| **Additional File 2: Table S2 Associations between TyG index and PCAT attenuation according to different diabetes statuses** | | | | | | |
| --- | --- | --- | --- | --- | --- | --- |
| **Characteristics** | Model 1 | | Model 2 | | Model 3 | |
|  | β (95% CI) | *P* value | β (95% CI) | *P* value | β (95% CI) | *P* value |
| Normoglycemia (n= 241) |  |  |  |  |  |  |
| TyG index (per SD) | 1.679 (-0.114, 3.473) | 0.067 | 2.038 (0.279, 3.798) | 0.023 | 1.405 (-0.401, 3.211) | 0.127 |
| TyG index (median)* |  | |  | |  | |
| Low level | 1 (Reference) | | 1 (Reference) | | 1 (Reference) | |
| High level | 1.595 (-1.258, 4.448) | 0.273 | 1.963 (-0.846, 4.772) | 0.171 | 1.154 (-1.711, 4.020) | 0.430 |
| Pre-diabetes mellitus (n=88) |  |  |  |  |  |  |
| TyG index (per SD) | 3.157 (0.795, 5.519) | 0.009 | 3.089 (0.531, 5.646) | 0.018 | 2.652 (0.060, 5.245) | 0.045 |
| TyG index (median)* |  | |  | |  | |
| Low level | 1 (Reference) | | 1 (Reference) | | 1 (Reference) | |
| High level | 4.624 (0.966, 8.282) | 0.013 | 4.953 (1.123, 8.782) | 0.011 | 4.689 (0.913, 8.465) | 0.015 |
| Diabetes mellitus (n=240) |  |  |  |  |  |  |
| TyG index (per SD) | 1.743 (0.613, 2.874) | 0.002 | 1.930 (0.750, 3.109) | 0.001 | 1.825 (0.643, 3.007) | 0.002 |
| TyG index (median)* |  | |  | |  | |
| Low level | 1 (Reference) | | 1 (Reference) | | 1 (Reference) | |
| High level | 3.769 (1.297, 6.242) | 0.003 | 3.986 (1.474, 6.497) | 0.002 | 3.675 (1.152, 6.198) | 0.004 |
| * TyG index was converted into a binary variable according to the median value.  Model 1: unadjusted;  Model 2: adjusted for age and sex;  Model 3: adjusted for age, sex, BMI, smoking, hypertension, eGFR, antiplatelet drug use, statin drug use, ACEI/ARB drug use, beta-blocker drug use, and antidiabetic drug use.  TyG, triglyceride-glucose; PCAT, pericoronary adipose tissue; CI, confidence interval; SD, standard deviation; BMI, body mass index; eGFR, estimated glomerular filtration rate; ACEI, angiotensin-converting enzyme inhibitor; ARB, angiotensin receptor blocker. | | | | | | |

| **Additional File 2: Table S3 Association of TyG index or PCAT attenuation with severity of CAD** | | | | | | |
| --- | --- | --- | --- | --- | --- | --- |
| **Characteristics** | Model 1 | | Model 2 | | Model 3 | |
|  | OR (95% CI) | *P* value | OR (95% CI) | *P* value | OR (95% CI) | *P* value |
| TyG index (per SD) | 1.248 (1.038, 1.500) | 0.019 | 1.244 (1.023, 1.514) | 0.029 | 1.165 (0.952, 1.424) | 0.138 |
| TyG index |  | |  | |  | |
| Low level (< 8.59) | 1 (Reference) | | 1 (Reference) | | 1 (Reference) | |
| High level (≥ 8.59) | 1.757 (1.231, 2.509) | 0.002 | 1.692 (1.158, 2.474) | 0.007 | 1.609 (1.093, 2.369) | 0.016 |
| PCAT attenuation (per SD) | 1.583 (1.314, 1.090) | < 0.001 | 1.545 (1.271, 1.878) | < 0.001 | 1.509 (1.238, 1.838) | < 0.001 |
| PCAT attenuation |  | |  | |  | |
| Low level (< -80.9) | 1 (Reference) | | 1 (Reference) | | 1 (Reference) | |
| High level (≥ -80.9) | 2.060 (1.416, 2.998) | < 0.001 | 1.876 (1.271, 2.769) | 0.002 | 1.807 (1.221, 2.674) | 0.003 |
| *P* value for interaction | - | | - | | 0.116 | |
| Model 1: unadjusted;  Model 2: adjusted for age, sex, BMI, smoking, hypertension, eGFR, antiplatelet drug use, statin drug use, ACEI/ARB drug use, beta-blocker drug use, and antidiabetic drug use;  Model 3: adjusted for age, sex, BMI, smoking, hypertension, eGFR, antiplatelet drug use, statin drug use, ACEI/ARB drug use, beta-blocker drug use, and antidiabetic drug use. TyG index was additionally adjusted for PCAT attenuation, and PCAT attenuation was additionally adjusted for TyG index.  PCAT, pericoronary adipose tissue; CAD, coronary artery disease; TyG, triglyceride-glucose; OR, odds ratio; CI, confidence interval; SD, standard deviation; BMI, body mass index; eGFR, estimated glomerular filtration rate; ACEI, angiotensin-converting enzyme inhibitor; ARB, angiotensin receptor blocker. | | | | | | |
